# Evaluation of UV photofunctionalization effect on ultrastructural properties of SLA titanium disks: An in vitro study

**DOI:** 10.34172/japid.2023.015

**Published:** 2023-09-11

**Authors:** Behzad Houshmand, Zeinab Rezaei Esfahroodi, Aliasghar Behnamghader, Sadegh Mohammadreza, Aytan Azizi, Kave Ramezani

**Affiliations:** ^1^Department of Periodontics, Faculty of Dentistry,Shahid Beheshti University of Medical Sciences, Tehran, Iran; ^2^Research Department of Nano-Technology and Advanced Materials, Karaj, Iran; ^3^Department of Periodontics, Faculty of Dentistry, Qazvin University of Medical Sciences, Qazvin, Iran; ^4^Department of Endodontics, Faculty of Dentistry, Qazvin University of Medical Sciences, Qazvin, Iran

**Keywords:** Dental implants, Photofunctionalization UV, Titanium disks

## Abstract

**Background.:**

The success rate of dental implants diminishes over time; the lack of osseointegration and infection are the major causes of most implant failures. One of the effective methods to improve the surface properties is to irradiate ultraviolet (UV) light. This study investigated the effect of UV photofunctionalization on the ultrasuperficial properties of sandblasted, large-grit, acid-etched (SLA) titanium discs.

**Methods.:**

In this in vitro study, 24 sandblasted and acid-etched titanium discs, with a lifespan of more than four weeks, were categorized into three groups (n=8): control, ultraviolet C (UVC), and ultraviolet B (UVB). Then, they were exposed to a UV light source for 48 hours at a 1-cm distance. In addition to measuring the contact angle between the liquid and the disc surface in each of the three groups, the atomic concentrations of carbon, oxygen, and nitrogen atoms were measured at three different sites on each disc. One-way ANOVA and post hoc Tukey tests were used to analyze data.

**Results.:**

The mean concentration of carbon atoms significantly differed in the control, UVC, and UVB groups (*P*<0.001). The mean concentrations of nitrogen atoms differed significantly between the three groups (*P*<0.001). However, the mean concentrations of oxygen atoms were not significantly different between the three groups. In examining the contact angle, wettability was higher in the UVC group than in the UVB group and higher in the UBV group than in the control group.

**Conclusion.:**

Photofunctionalization with UV light significantly decreased carbon and nitrogen concentrations on the surface of titanium implants, indicating that the implant’s superficial hydrocarbons were eliminated. It was observed that UVC photofunctionalization was more effective than UVB photofunctionalization in reducing superficial contamination and improving wettability.

## Introduction

 With a 98% initial success rate, dental implants have evolved into a common and effective treatment option for missing teeth. This success rate, nevertheless, gradually declines as infection and a lack of osseointegration become the main reasons for implant failures.^1^

 Titanium is a transition metal with high strength and low density, which is compatible with human tissues and can be slowly integrated into the bone tissue, known as osseointegration.^2^ These properties diminish on surfaces with a lifespan of more than 2‒4 weeks, known as biological aging. It is important because the restoration time of implants fabricated more than four weeks earlier is twice that of newly fabricated implants.^3-5^

 One of the effective methods to improve the superficial properties is photofunctionalization, involving ultraviolet (UV) irradiation with a wavelength of 100‒400 nm without changes in the irradiated surface topography. UV wavelengths of 320‒400, 290‒320, and 100-290 nm are categorized as ultraviolet A (UVA), ultraviolet B (UVB), and ultraviolet C (UVC), respectively.^6^ Photofunctionalization with UV radiation leads to favorable changes (chemical composition and electric charge) and improved biological properties of the surface of titanium implants. These changes occur without differences in favorable topographic properties for osseointegration, where UV irradiation could increase the success rate of the implant to about 100% by accelerating and completing osseointegration.^7,8^

 The most suitable commercially available titanium surface for dental implants is the sandblasted, large-grit, acid-etched (SLA) surface, which has nano-pits and micro-pits. When UV light is used to photofunctionalize the surface of SLA, the implant’s contact area with bone grows, protein adhesion and absorption increase, mesenchymal cells differentiate into the osteoblast line, and osteogenic cells proliferate, differentiate, and become mineralized.^8,9^

 Photofunctionalization can break down weak bonds between contributing hydrocarbons and titanium, which can cleanse the titanium surface in some way so that the space required for reactions between O, N, and S atoms of the tissue molecules and titanium would not be occupied.^10-12^

 Dini et al^13^ examined the application of photofunctionalization with UV radiation on dental implants. They concluded that UV exposure decreased hydrocarbon content and increased blood plasma proteins in humans and albumin absorption across sample surfaces. These samples had higher wettability, causing diminished microbial activity. Mehl et al^14^ examined the effect of photofunctionalization with UV radiation on the osseointegration of titanium dental implants in swine mandibles with UV irradiation before implantation. After nine months, no significant effect was found in the osseointegration and stability of the titanium implant.

 Even in irradiated samples after four weeks, contamination with hydrocarbons and a decrease in surface biological characteristics are seen because biological aging lowers the success rate of implants.^15,16^ Thus, approaches should be adopted to enhance osseointegration and prevent bacterial colonization to achieve more reliable and durable treatment. Thus, this study investigated the effect of UV photofunctionalization on the ultrastructural properties of SLA titanium disks.

## Methods

**Figure 1:**
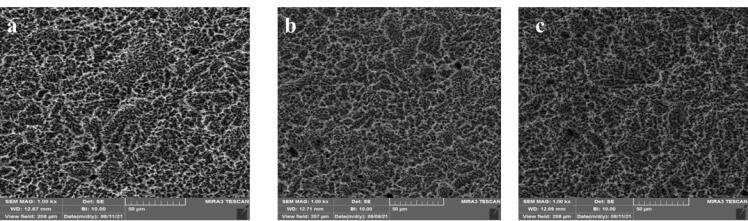
SEM images of the analyzed region of atomic concentration of superficial elements in the three groups; (a) control, (b) UVC, and (c) UVB. (Extension = png, Width = 658, Height = 273, Resolution = 600*600)

**Figure 2:**
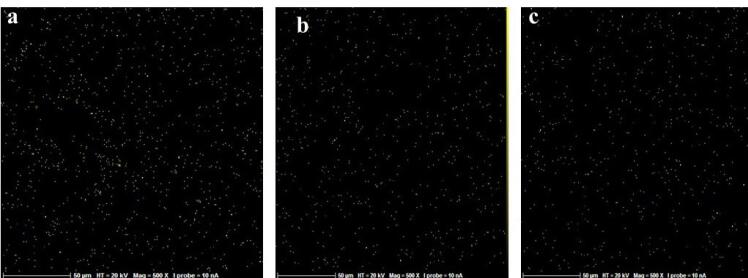
Map scan for examining the dispersion of carbon atom across disk in all the three groups; (a) control, (b) UVC, and (c) UVB. (Extension = png, Width = 658, Height = 273, Resolution = 600*600)

**Figure 3:**
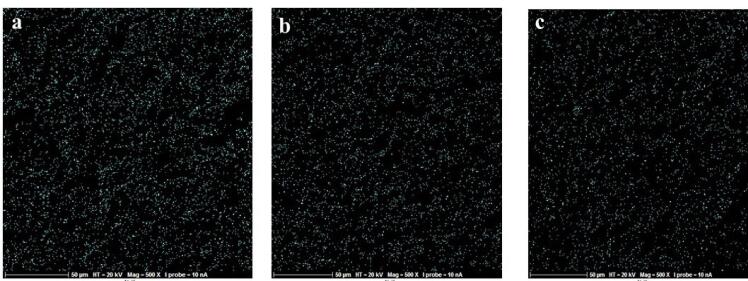
Map scan for examining the dispersion of nitrogen atoms across the disk in all the three groups; (a) control, (b) UVC, and (c) UVB. (Extension = png, Width = 658, Height = 273, Resolution = 600*600)

 In this in vitro study, 24 sandblasted and acid-etched commercial titanium disks (Biotem, Korea) with a diameter of 8 and a thickness of 2 mm were chosen. The disks had been synthesized more than four weeks earlier. The titanium disks were placed in three groups (n = 8): group I) the control group containing intact disks; group II) the disks that underwent UVC irradiation; and group III) the disks that underwent UVB irradiation. For UV photofunctionalization, they underwent UVC irradiation at 20-W power and an approximate wavelength of 210 nm (Phillips, the Netherlands). UVB had 20-W power and an approximate wavelength of 310 nm (Phillips, the Netherlands). The titanium disks of UVB and UVC groups were separately placed 1 cm away from the light source for 48 hours. At a distance of 1 cm from the light source, the energy reaching the sample was measured using a Wattmeter instrument (TES Electrical Electronic Corp, Taiwan). This value was 6 W for the UVC group and 3.7 W for the UVB group. From each of the groups (control, UVC, and UVB), three sandblasted and acid-etched commercial disks were utilized for energy dispersive spectroscopy (EDS) analysis. The samples were transferred to the laboratory in closed containers in the dark. EDS analysis was performed on five different points of each disk using SEM (TeScan-Mira III Czech). The disks were analyzed with an electron acceleration of 20 kV within an approximate 200-mcm range. The weight percentage of oxygen, nitrogen, and carbon atoms was studied in these regions (Figures 1, 2, and 3).

**Figure 4:**
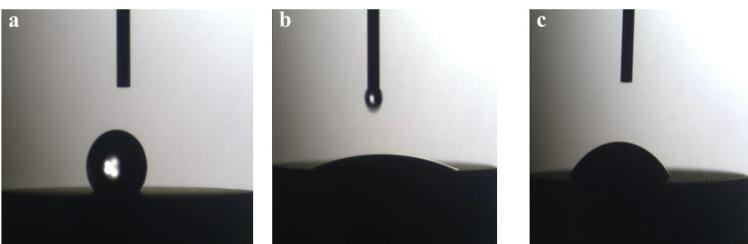
Wettability test in all the three groups; (a) control, (b) UVC, and (c) UVB. (Extension = png, Width = 658, Height = 273, Resolution = 600*600)

 The wettability of titanium disks was studied by the Sessile Drop method with the help of the SCAM-S1 instrument from the MNT (MehrTavaNegar Alborz, Iran) Research Company. A software-based image analyzer was used for analyses. The contact angle was measured after placing four microliters of deionized water on a surface. Using this device, the contact angle of liquids on the surface of solid materials can be calculated visually.^17^ When measuring the contact angle for the UVC group, when the water drop affected the disk’s surface, the water drop was immediately dispersed across the disk surface and created an angle of 0°. However, for measuring the contact angle and recording the image, the image of the first effect of water drop on the disks’ surface within the first second was recorded and set as the basis for measuring the contact angle in the three groups (Figure 4).

 The data were analyzed by one-way ANOVA, Tukey post hoc tests, or the Games-Howell test using SPSS 26.

## Results

 Twenty-four SLA titanium disks were categorized into three groups (n = 8), including control, UVC, and UVB, in which the atomic concentrations were measured for carbon, oxygen, and nitrogen at three points of each disk alongside the contact angle of the liquid against the surface for all three groups.

 Comparisons of mean carbon atom concentrations between the three groups indicated significant differences between the three groups (*P* < 0.001; Table 1). In the paired comparison of the groups, carbon atom concentration in the UVC group was significantly different from the UVB and control groups (*P* < 0.001); it was significantly lower in the UVC group than in the control and UVB groups. However, carbon atom concentration in the UVB group was not significantly different from the control group (*P* = 0.171).

**Table 1:** Comparison of mean concentrations of carbon, nitrogen, and oxygen atoms in UVB, UVC, and control groups

**Groups**	**UVC**	**UVB**	**Control**	***P***** value**
	**Mean±SD**	**Mean±SD**	**Mean±SD**	
Carbon	0.75 ± 0.02	0.90 ± 0.07	0.95 ± 0.07	< 0.001
Nitrogen	3.92 ± 0.25	4.12 ± 0.76	4.17 ± 0.25	< 0.001
Oxygen	4.62 ± 0.32	4.82 ± 0.29	4.97 ± 52	0.64

 Comparisons of the mean nitrogen atom concentration in the three groups showed significant differences (*P* < 0.001) between the three groups (Table 1). In the pairwise comparisons of the groups, nitrogen atom concentration in the UVC group was significantly different from the UVB (*P* = 0.047) and control (*P* < 0.001) groups; it was significantly lower in the UVC group than in the two other groups. However, nitrogen atom concentration in the UVB group was not significantly different from the control group (*P* = 0.785).

 Comparisons of the oxygen atom concentrations between the three groups showed no significant differences between the three groups (*P* = 0.064) (Table 1).

 Comparisons of the mean contact angles showed significant differences between the three groups (*P* < 0.001). Pairwise comparisons of the groups showed that the contact angle was significantly lower in the UVC group compared to the UVB and control groups (*P* < 0.001). The contact angle was significantly lower in the UVB group compared to the control group (*P* < 0.001) (Table 2).

**Table 2:** Comparison of the contact angles in UVB, UVC, and control groups

**Groups**	**No.**	**Mean±SD**	***P***** value**
UVC	15	16.40 ± 0.54	< 0.001
UVB	15	58.92 ± 0.43	
Control	15	129.88 ± 0.70	

## Discussion

 According to the present study’s findings, carbon and nitrogen atom concentrations in the UVC group were significantly lower than those in the UVB and control groups, whereas the concentrations of oxygen atoms did not vary significantly between the three groups. Henningsen et al^18^ compared the effect of applying UV radiation on non-thermal plasma treatment concerning improvements in the physical properties and cellular reactions across the titanium surface. They found that UV irradiation significantly impacted the contact angle of titanium disks,^15^ where the thickness of the titanium oxide layer after UV irradiation increased significantly; however, this increase was lower compared to surface modification with the plasma spraying method. Additionally, UV functionalization significantly impacted carbon atoms on the titanium disks’ surface, consistent with the current study’s findings. The distinction is that although Henningsen et al^18^ examined the effects of many UV radiation spectra, the current work examined the effects of each spectrum individually. In a study by Hori et al,^19^ 4-week titanium disks undergoing treatment with UV radiation showed higher protein absorption, osteoblast migration, adhesion, differentiation, and mineralization compared to the freshly produced disk surfaces, and their extent of hydrophilicity dramatically increased. Thus, the mentioned biological changes contribute to reducing the hydrocarbons of the titanium surface following treatment with UV radiation.

 In examining changes in the surface elements of dental implants following UV photofunctionalization, as well as variations in the surface atoms of oxygen, nitrogen, and carbon, Roy et al^5^ found that after titanium surface exposure to the atmosphere, the titanium oxidized surface could bind to hydrocarbon contaminants through reaction with carboxyl and amine groups. The biological aging process was accelerated when there was a rise in the concentration of these hydrocarbons on the implant surface. Additionally, the fraction of carbon and nitrogen atoms decreased significantly following UV exposure, whereas the percentage of oxygen atoms increased to some extent. Based on the model of Roy et al,^5^ long-term exposure to UV radiation photons can cause dissociation of the bond between hydrocarbons and titanium atom at the implant surface, as well as reduction of H_2_O level on the implant surface. The present study is consistent with Roy and colleagues’ research regarding carbon and nitrogen reduction.

 Photofunctionalization via changing the electric charge of the implant surface leads to enhanced adhesion to surrounding tissues.^20,21^ Meanwhile, TiO_2_ exposure to UV radiation leads to electronic excitation from the capacity band to the conduction band, making the TiO_2_ surface electrically positive.^22^ Aita et al^15^ studied machined and rough titanium surfaces and showed that after exposure of titanium surfaces to UV radiation, superficial hydrocarbons were removed, and the titanium oxide layer increased.

 Elsewhere, in examining the effect of UV photofunctionalization on machined implants against SLA implants, Lee et al^7^ observed that the carbon content in the machined and SLA disks decreased after UV radiation, consistent with the present study.

 The duration of the radiation, its intensity and wavelength, and the distance between the light source and the sample may all impact how much the UV radiation alters the produced alterations.^23^ Gao et al^24^ compared the effects of UVC and UVA radiations on the biological activity of the surface of titanium implants after exposure for 24 hours, concluding that UVA and UVC treatments reduced hydrocarbon levels, where UVC radiation functioned more powerfully. Nevertheless, because of minor effects, UVA was not examined.

 Surface wettability plays a significant role in the regeneration and restoration of the host tissue cells near the implant surface. It is mainly dependent on the free energy of the surface. The interface between the implant surface and biological media may be improved by superficial wettability. Additionally, wettability promotes the uptake of proteins and the adhesion and proliferation of cells. It is affected by the surface chemistry and topographic parameters, such as roughness and microstructure. Titanium surfaces with higher superficial energy and wettability may establish a greater ability to induce osteoblast differentiation. Thus, measuring the superficial energy can be a predictive index for cellular compatibility.^25^ Positive chemical changes in the surface and hydrophilicity, including a considerable reduction in carbon residues following UV radiation, even in dental implants based on acid-etched zirconia, were observed in a study by Tuna et al.^26^

 Wettability as an index of superficial energy grows with UV irradiation.^27^ The surface nano-structure and chemical composition determine the extent of wettability, further characterizing the initial events and biological cascade across the biomaterial/host binding surface.^28^

 Based on the contact angle between water drops and the surface, surfaces are categorized into three groups: super-hydrophilic (contact angle of 0°), hydrophilic (contact angle < 90°), and hydrophobic (contact angle > 90°). According to the findings of the current investigation, the UVC group’s contact angle was much lower than that of the control and UVB groups. Additionally, the contact angle in UVB was significantly lower compared to the control. The UVC group showed very hydrophilic characteristics. In the UVB group, no scattering of the water drop was observed after impacting the surface, and the water drops remained unchanged on the surface. Thus, it can be concluded that UVB irradiation successfully converted the surface of disks to a hydrophilic surface, but it could not create a super-hydrophilic surface, which can be attributed to the lower ability of UVB irradiation to reduce the carbon level of the surface compared to UVC. The contact angle in the control group indicated that the titanium disks that had undergone biological aging exhibited a hydrophobic surface.

 The extent of hydrophilicity of titanium implants after treatment with UV irradiation increases dramatically.^15^ Again, the results of the wettability test and contact angle measured for each group enhanced the wettability of titanium implants after UV photofunctionalization.

 Considering the significant effect of UV photofunctionalization on the chemical and physical properties of the implant surface, it was found that UVC radiation, as compared to UVB, had a greater effect on these properties. As such, UVC is better for applied and research purposes so that the maximum effect of this radiation can be evaluated and harnessed on the superficial properties of implants.

 The study’s limitations include the absence of stronger radiation sources, the inaccessibility of more sophisticated surface analyses like XPS, the study’s laboratory setting, certain differences from clinical circumstances, and its confinement to a single exposure period.

## Conclusion

 Photofunctionalization with UV irradiation significantly decreased the concentrations of carbon and nitrogen atoms on titanium implant surfaces, suggesting the elimination of the superficial hydrocarbons of the implant. Moreover, its effect on improving the biological properties and wettability was justified. UVC photofunctionalization was more effective than UVB photofunctionalization in reducing superficial contamination and improving the wettability properties.

## Competing Interests

 The authors declare that they have no competing interests with regard to the authorship and/or publication of this article.

## Ethical Approval

 The protocol of the present study was approved by the Ethics Committee of Shahid Beheshti University of Medical Sciences under the code IR.SBMU.DRC.REC.1399.147.

## Funding

 None.
